# A131 ENDOSCOPIC SUBMUCOSAL DISSECTION: EXPERIENCE AT A LOCAL CANADIAN CENTER

**DOI:** 10.1093/jcag/gwae059.131

**Published:** 2025-02-10

**Authors:** C Ching Hui Yee, R Bechara

**Affiliations:** Division of Internal Medicine, McGill University Faculty of Medicine and Health Sciences, Montreal, QC, Canada; Kingston Health Sciences Centre, Kingston, ON, Canada

## Abstract

**Background:**

Endoscopic Submucosal Dissection (ESD) was established in Japan. There exist few formalized training programs compared to the growing need for its expertise internationally, with many learning through observation and in-vivo training courses. According to Oyama et. al. (1), western centers conducting ESD have learning curves which were inferior when compared to leading Japanese centers. The proposed quality metrics for a proficient operator in ESD are en bloc resection rates of 90%, complications <5%, curative resection rates of 80%, and resection speed of 9cm2/hr. Whereas, the proposed quality metrics for competency are en bloc resection rates of 80% and complication rates of <10%. (1) Here, we present ESD data from a local Canadian center performed by an expert who underwent formal training in ESD at a expert Japanese center.

**Aims:**

We aim to assess if internationally proposed quality metrics of an operator proficient in ESD were met.

**Methods:**

We retrospectively reviewed all patients who underwent ESD between October 2016 up till September 2024 at the Kingston Health Sciences Center. 324 consecutive ESDs were performed on the esophagus, stomach, duodenum, colon and rectum. Primary outcomes include resection speed calculated as centimeters squared per hour (cm2/hr). Secondary outcomes include the number of successful en block and R0 resections, total procedure time, and procedure-related adverse events. Demographic and procedural variables were compared with descriptive statistics (mean ± SD; median, interquartile range), two-sample t-test, and chi-square test.

**Results:**

324 (62.7% male) (67.6 ± 11.8 years) consecutive ESDs were performed, consisting of esophageal (n=109), gastric (n=55), duodenal(n=1), rectal (n=80), and colonic (n=79) ESDs. The overall mean resection speed was 10.9 ± 6.9 cm2/hr. The rate of successful en bloc resections was 96%, and R0 resections were 88.6%. Curative resection rates were 82.4%. The total procedure-related adverse events were at 4%, Notably, 1 of the perforations were delayed, requiring surgical intervention.

When the ESD data was divided into early (first 160 cases) and late (later 164 cases) groups, there was a significant improvement in the resection speed (10.1 ± 7.1 cm2/hr vs 11.7 ± 6.7 cm2/hr, p = 0.05), while maintaining a consistently high rate of en bloc resection (94.4% vs 98.2%) and a low rate of adverse events (4.4% vs 3.7%).

**Conclusions:**

This study demonstrates that ESDs conducted after formal training not only met proficiency quality metrics from the outset, but also exhibited improvements in resection speed and en bloc resection rates as experience increased.

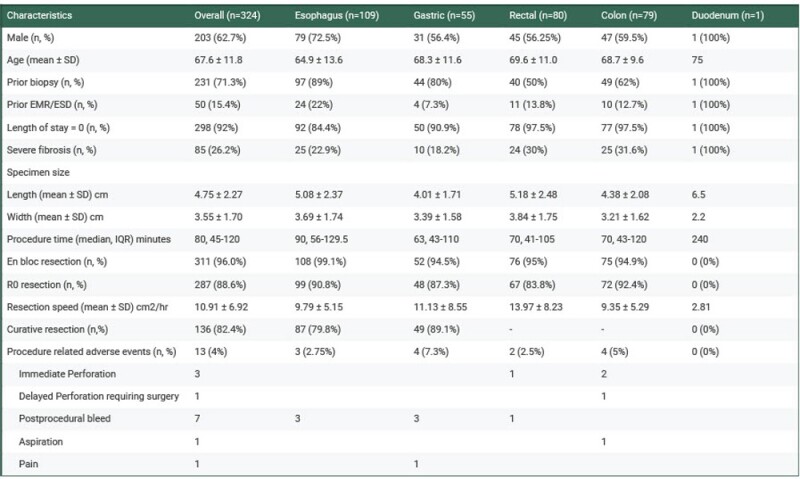

Characteristics of ESD

**Funding Agencies:**

None

